# Transcranial direct current stimulation for the treatment of motor impairment following traumatic brain injury

**DOI:** 10.1186/s12984-019-0489-9

**Published:** 2019-01-25

**Authors:** Won-Seok Kim, Kiwon Lee, Seonghoon Kim, Sungmin Cho, Nam-Jong Paik

**Affiliations:** 1 Department of Rehabilitation Medicine, Seoul National University College of Medicine, Seoul National University Bundang Hospital, 82, Gumi-ro 173 Beon-gil, Bundang-gu, Seongnam-si, Gyeonggi-do 13620 Republic of Korea; 2 Ybrain Research Institute, Seongnam-si, Republic of Korea; 3 Delvine Inc., Seoul, Republic of Korea

**Keywords:** Traumatic brain injuries, Transcranial direct current stimulation, Recovery of function, Rehabilitation, Neuronal plasticity, Electroencephalography, Functional near infrared spectroscopy, Virtual reality

## Abstract

After traumatic brain injury (TBI), motor impairment is less common than neurocognitive or behavioral problems. However, about 30% of TBI survivors have reported motor deficits limiting the activities of daily living or participation. After acute primary and secondary injuries, there are subsequent changes including increased GABA-mediated inhibition during the subacute stage and neuroplastic alterations that are adaptive or maladaptive during the chronic stage. Therefore, timely and appropriate neuromodulation by transcranial direct current stimulation (tDCS) may be beneficial to patients with TBI for neuroprotection or restoration of maladaptive changes.

Technologically, combination of imaging-based modelling or simultaneous brain signal monitoring with tDCS could result in greater individualized optimal targeting allowing a more favorable neuroplasticity after TBI. Moreover, a combination of task-oriented training using virtual reality with tDCS can be considered as a potent tele-rehabilitation tool in the home setting, increasing the dose of rehabilitation and neuromodulation, resulting in better motor recovery.

This review summarizes the pathophysiology and possible neuroplastic changes in TBI, as well as provides the general concepts and current evidence with respect to the applicability of tDCS in motor recovery. Through its endeavors, it aims to provide insights on further successful development and clinical application of tDCS in motor rehabilitation after TBI.

## Background

Traumatic brain injury (TBI) is defined as “an alteration in brain function (loss of consciousness, post-traumatic amnesia, and neurologic deficits) or other evidence of brain pathology (visual, neuroradiologic, or laboratory confirmation of damage to the brain) caused by external force” [1]. The incidence and prevalence of TBI are substantial and increasing in both developing and developed countries. TBI in older age groups due to falling has been on the rise in recent years, becoming the prevalent condition in all age groups [2, 3]. TBI causes broad spectrum of impairments, including cognitive, psychological, sensory or motor impairments [4, 5], which may increase the socioeconomic burdens and reduce the quality of life [6, 7]. Although motor impairment, such as limb weakness, gait disturbance, balance problem, dystonia or spasticity, is less common than neurocognitive or behavioral problems after TBI, about 30% of TBI survivors have reported motor deficits that severely limited activities of daily living or participation [8].

Motor impairment after TBI is caused by both focal and diffuse damages, making it difficult to determine the precise anatomo-clinical correlations [9, 10]. According to previous clinical studies, recovery after TBI also seems worse than that after stroke, although the neuroplasticity after TBI may also play an important role for recovery [11]. Therefore, a single unimodal approach for motor recovery, including conventional rehabilitation, may be limiting, and hence, requiring a novel therapeutic modality to improve the outcome after TBI.

Transcranial direct current stimulation (tDCS) – one of the noninvasive brain stimulation (NIBS) methods – can increase or decrease the cortical excitability according to polarity (anodal vs. cathodal) and be used to modulate the synaptic plasticity to promote long-term functional recovery via long-term depression or potentiation [12, 13]. Recent clinical trials evaluating patients with stroke have reported the potential benefits of tDCS for motor recovery [14]. Neuroplastic changes after TBI and results from animal studies also suggest that tDCS could improve the motor deficit in TBI, although clinical trials using tDCS for motor recovery in TBI are currently lacking [14].

In this review, we will cover (1) the pathophysiology and possible neuroplastic changes in TBI; (2) physiology of tDCS; (3) current clinical evidence of tDCS in TBI for motor recovery; (4) general current concept of tDCS application for motor recovery; and (5) the future developments and perspectives of tDCS for motor recovery after TBI. Although the scope of motor recovery is wide, this review will focus primarily on the recovery of limb function, especially that of the upper limb. We expect that this review can provide insights on further successful development and clinical application of tDCS in motor rehabilitation after TBI.

## Pathophysiology and possible neuroplastic changes after TBI

### Acute stage

#### Primary injury

According to the mechanism of trauma, there can be various types of focal injury. Penetration can directly damage the brain tissue and blood vessels, leading to intracranial hemorrhage. Direct blow can cause coup and countercoup injury of the brain parenchyma. Cerebral contusion caused by non-contact external force or countercoup is common in the temporal or frontal lobes due to the fragile surface being vulnerable to the sharp and rough edges of the anterior and middle cranial fossa [15]. The acceleration-deceleration force from the trauma can cause diffuse axonal injury by the strain, translational or rotational forces. The commonly involved white matter areas by the diffuse axonal injury are the brainstem, corpus callosum, basal ganglia, thalamus, and cerebral hemispheres [16]. Despite the small focal injury, the accompanying diffuse axonal injury may cause severe functional impairment due to the loss of connectivity between the functionally-connected areas [17].

#### Secondary injury

**Fig. 1:**
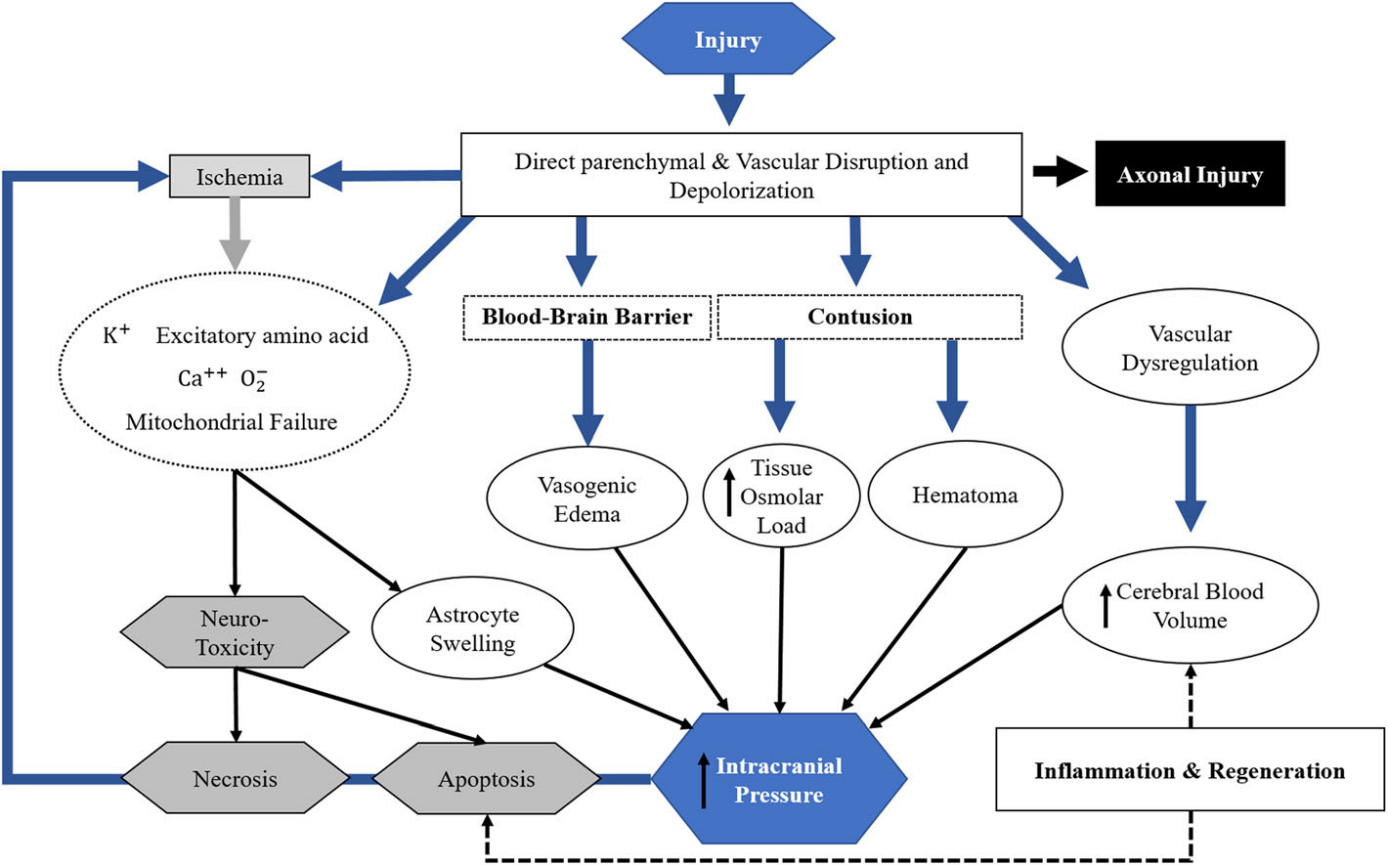
Pathophysiologic mechanisms of secondary injury after traumatic brain injury (Figure modified from reference [25])

Secondary injury occurs any time from immediately following the primary injury to several weeks after the primary injury and can be caused by the following possible mechanisms: excitotoxicity, cerebral edema, ischemia, and neuro-inflammation (Fig. 1). In brief, an increase in the release of glutamate induces the influx of calcium ion into the neuronal cells, causing a series of harmful effects. These serial changes include exacerbated metabolic stress, mitochondrial damage [18], accumulation of reactive oxygen species [19], calcium-induced calpain proteolysis [20], and activation of endothelial and neuronal nitric oxide synthetase, which leads to increased nitric oxide [19]. Both vasogenic edema caused by blood brain barrier disruption and cytotoxic edema caused by neuronal cell dysfunction or death aggravates the degree of injury [21]. Direct vascular and blood brain barrier disruption interferes with blood flow autoregulation and decreased perfusion, potentially leading to cerebral ischemia [22]. Acute inflammation may occur after TBI, which is mediated by neutrophils, macrophages, and pro-inflammatory cytokines, contributing to further secondary damages as well as tissue regeneration and plasticity [23, 24]. Kochanek et al. provides a comprehensive review of secondary injuries after TBI [25].

### Subacute stage

After acute injury, remyelination or neuroplasticity contributes to motor recovery, which is most eminent within the first 3 months after injury [26]. In the subacute state, GABA-mediated inhibition seems to play an important role in neuroplasticity. Although an increase in the GABA-mediated intervention may be beneficial during the acute phase [27], continued increase can interfere with recovery. Kobori et al. demonstrated that increased GABA levels are associated with long-term memory impairment, which may be restored after the administration of GABA antagonists [28]. O’Dell et al. also reported similar results supporting that the modulation to decrease the GABA-medicated inhibition could promote recovery [29, 30]. Both animal and human studies regarding stroke also demonstrated the important role of GABA-mediated inhibition on the motor recovery [31, 32]. Therefore, the intervention to modulate GABAergic activity may be promising for motor recovery in subacute stage of TBI.

### Chronic stage

After sustaining brain damage, neuroplastic changes could either be adaptive or maladaptive, the latter may be associated with poor functional recovery. In a previous study of patients with stroke, both contralesional and ipsilesional motor cortices were activated during voluntary movement of the paretic hand [33]. When patients recovered poorly, the activation of contralesional motor cortex was greater, and these neuroplastic changes are now considered as maladaptive neuroplastic changes [34, 35]. Therefore, NIBS for the modulation of maladaptive plasticity, even during the chronic stages, could be beneficial [36].

## Physiology of tDCS

tDCS delivers direct constant electrical currents to the cortical area of brain between two electrodes (anode and cathode), modulating the neuronal excitability by changing the resting membrane potential level [37]. The change in the direction of excitability after tDCS mainly depends on the electrode montages [38]. For instance, an anodal stimulation over the motor cortex increases the excitability, whereas a cathodal stimulation decreases the excitability [39]. Short-term effects of tDCS appear to be caused by alterations in hydrogen ions and transmembrane proteins, which is a nonsynaptic mechanism [40]. The long-term effects of tDCS may depend on synaptic modulation, which is long-term potentiation or long-term depression [38, 41]. Anodal tDCS could induce long-term potentiation by modulating GABA_A_ergic and glutamatergic synapses [42, 43], whereas cathodal tDCS could induce the long-term depression by reducing the glutamatergic activity [44].

Therefore, according to various changes in different stages after TBI, different tDCS protocols can be considered [45]. Cathodal tDCS can be considered during the acute stage to decrease the glutamate-mediated excitotoxicity. In the subacute stage, anodal tDCS can be considered to reduce the GABA-mediated inhibition. Moreover, tDCS with behavioral interventions can be considered during the chronic stages to overcome maladaptive plasticity. These are only suggestions and future clinical trials are needed to prove the efficacy of tDCS and to define the optimal location for stimulation as well as the parameters associated with tDCS in patients with TBI.

## Current clinical evidence of tDCS in TBI for motor recovery

Although there have been studies investigating the effect of NIBS on the non-motor impairments (e.g. depression, memory, attention) in patients with TBI [14, 46], studies for motor recovery is lacking. In the study including only two patients with TBI, bi-hemispheric tDCS on C3 and C4 (1.5 mA for 15 min/session, total 24 sessions) improved the upper extremity Fugl-Meyer scores for up to 6 months after treatment [47]. Some recent animal studies with the TBI model have also been published. In a unilateral controlled cortical impact model, Jefferson et al. reported greater behavioral improvements and increased wrist motor cortical presentation after ipsilesional 100 Hz cortical stimulation with reaching training when compared with the reaching training only [48]. However, the overall degree of recovery was modest and less than the recovery level in similar stroke studies [49, 50], which may implicate that the parameters of cortical stimulation from stroke studies are suboptimal in moderate and severe TBI. Recovery and neuroplastic mechanism after TBI could be different from that after stroke [51], and a future study using tDCS to prove the efficacy and define the parameters for better recovery (e.g. stimulation location, mode, duration) in TBI is needed.

In a recent study with controlled cortical impact model, a standalone ipsilesional 30 Hz cortical stimulation demonstrated no significant behavioral improvements or lesion size difference using FDG-microPET when compared with no stimulation [52]. This result corresponds with the opinion of Talelli et al., who asserted that cortical stimulation alone could not induce the brain to from appropriate connections needed for recovery [53], implying that behavioral therapy must be combined with cortical stimulation for motor recovery.

Stroke causes motor impairment as a result of cortical or subcortical damages and motor recovery is associated with neuroplastic changes, which is similar with TBI [54]. Therefore, clinical studies evaluating tDCS in patients with stroke could provide implications for its applicability in TBI. Recent Cochrane review showed a positive effect of tDCS on activities of daily living performance compared with the sham intervention at the end of the intervention period and at the end of the 3-month follow-up period [36]. However, tDCS on the upper extremity function revealed no evidence of a better effect than the control. In a recent study using a network meta-analysis of randomized controlled trials, only cathodal tDCS demonstrated a positive effect on improving the activities of daily living capacity but arm function measured by the Fugl-Meyer upper extremity assessment was not improved by tDCS [55]. Therefore, the effect of tDCS on motor recovery is still modest even in patients with stroke and a well-designed study with a larger number of patients is needed.

## General current concept of tDCS application for motor recovery

**Fig. 2:**
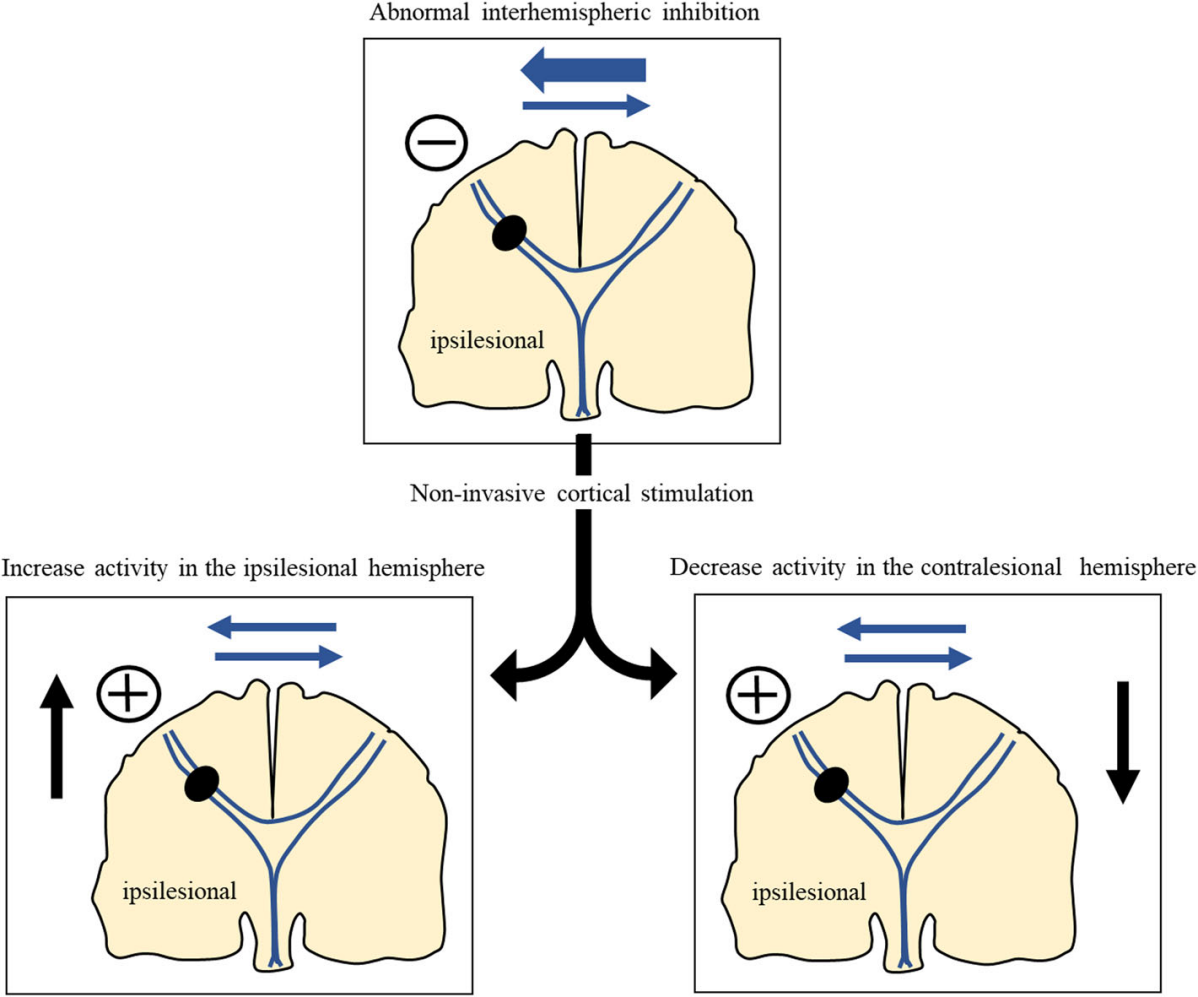
Strategy of noninvasive brain stimulation based on the interhemispheric inhibition model (Figure modified from reference [58])

Traditionally, the interhemispheric inhibition model was proposed to develop a strategy for neuromodulation after stroke. Although the pathophysiology could be different in patients with TBI, this concept may be applicable to those with TBI who have hemiparesis or hemiplegia due to the focal brain parenchymal lesion. In patients with stroke, the motor cortex activations in the bilateral hemispheres are counterbalanced by the interhemispheric inhibition [56]. The intact contralesional motor cortex will drive higher inhibitory signals to the ipsilesional motor cortex and then ipsilesional motor cortex will be over-inhibited (maladaptive plasticity), which will lead to poor motor recovery (Fig. 2) [57, 58]. Therefore, cathodal tDCS over the contralesional motor cortex to inhibit the over-inhibition of ipsileional motor cortex or direct excitation of ipsilesional motor cortex by anodal tDCS over the ipsilesional motor cortex can be considered for reducing the maladaptive plasticity (Fig. 2). However, the interhemispheric inhibition model is challenged, because this model is based on the studies only in the chronic and mild stroke patients [56, 59]. For example, in stroke patients with severe motor impairments due to extensive injury of corticospinal tract, increase in the activation of contralesional motor cortex might be important for the recovery [60–62]. Cathodal tDCS over the contralesional hemisphere induced no significant recovery in patients with extensive corticospinal tract damage, whereas it was effective in patients with small corticospinal tract damage [63]. Therefore, Pino et al. suggested the bimodal balance-recovery model, which is modulated by the degree of structural reserve [61]. If the structural reserve is high, interhemispheric inhibition model plays an important role in recovery. Conversely, if the structural reserve is low, the role of interhemispheric inhibition model is less important for recovery and the activation of contralesional hemisphere may play a more important role; hence anodal tDCS over the contralesional hemisphere may be beneficial. Further studies to prove this bimodal balance-recovery model in various stages (acute, subacute, chronic) and severities of stroke are needed for a more tailored tDCS protocol.

## Future development and perspective of tDCS for motor recovery after TBI

There are several concerns regarding the use of tDCS in a real clinical setting due to the associated intra- and inter-individual variabilities with respect to electrical current, responses and optimal stimulation target. Although tDCS offers greater convenience than magnetic stimulation, its accessibility to users, clinicians or patients, remains low. Appropriate task-oriented training must be implemented to augment the effect of tDCS for motor recovery [52, 53]. Therefore, further research and development of tDCS is necessary to address such limitations and to maximize the effect of tDCS on motor recovery after TBI.

### Personalized tDCS

Electrical current induced by tDCS is variable in accordance with the individual different head anatomy [64–67]. In addition, the intensity or distribution of current by tDCS could be modified in TBI patients with skull defect or skull plates after surgery [68]. Therefore, a personalized tDCS using MRI-based computational modeling could be an effective solution to overcome these limitations. The computational modeling techniques have widely been used to calculate the theoretic electric field induced by tDCS and optimize the electrode positions for the maximization of current intensity on the target areas with consideration to the unique head anatomy of each individual [64, 65].

**Fig. 3:**
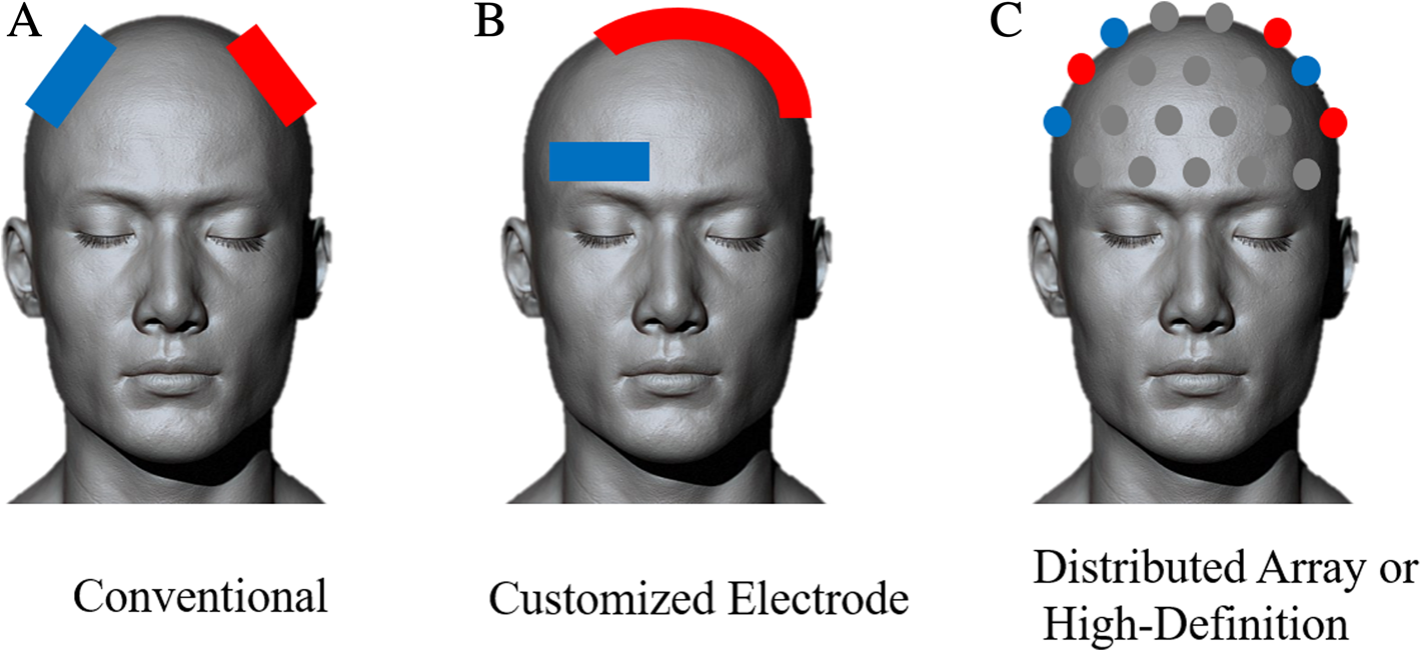
Schematic classification of personalized tDCS for motor recovery. Depending on electrode size, shape, and arrangement, tDCS can be broadly classified into **a** Conventional tDCS, **b** Customized Electrode tDCS, and **c** Distributed Array or High-Definition tDCS. Red color represents anodes and blue color represents cathodes

Moreover, recent advancements in the computational modeling have enabled a novel high-definition tDCS technique with manually configured array electrodes for relatively improved spatial resolution [65]. The effectiveness of the high-definition tDCS technique has been reported by showing increased motor evoked potential (MEP) amplitudes compared with those after conventional anodal tDCS stimulation on the primary motor cortex [69]. Figure 3 shows schematic classification of electrode arrays for personalized tDCS, which may more effectively and precisely modulate the focal area [66, 67].

#### Analysis of tDCS responses

If clinicians can monitor the tDCS responses before, during, and after stimulation, these changes can be used as surrogate markers for the effect of tDCS on neuroplasticity and the stimulation parameters could be adjusted according to these results. MEP can be one of the candidate surrogate markers reflecting immediate changes in the brain function by tDCS [12]. During the multiple sessions of anodal tDCS, MEP response to one anodal tDCS session may predict the response to subsequent sessions [70]. These results indicate that measurement of immediate functional responses of the brain by MEP after tDCS can be useful in monitoring the efficacy of tDCS.

Recent advancements in software-based signal processing techniques have enabled rapid or real-time analyses of functional activation of the brain [71–76]. Integration of these techniques into the tDCS system may improve the efficacy in a real-clinical setting. Functional magnetic resonance imaging (fMRI) can be used to monitor the functional changes induced by tDCS [71]. However, accessibility for fMRI is limited due to space, cost, complex signal processing, and low temporal resolutions to monitor the immediate blood oxygen level-dependent signal changes; hence real-time application may be difficult.

Electroencephalography (EEG) can reflect the tDCS-induced immediate changes in functional activation and networks in the brain. tDCS increased the 8-13 Hz mu event-related desynchronization, which showed a direct correlation with motor threshold [73]. Anodal tDCS over the primary motor cortex increased the functional connectivity in the premotor, motor, and sensorimotor areas during motor tasks [74]. These findings demonstrate that consistent and predictable changes measured by EEG can be used to monitor or evaluate immediate responses after tDCS. EEG has advantages, including high temporal resolution [77], that provide various possible information associated with the effect of tDCS (e.g. power spectrum, event-related potentials, coherence) [78]. EEG with dry electrodes having the acceptable impedance level could improve the usability in a real clinical setting [79]. However, in case of simultaneous EEG-tDCS use, the EEG signal should be carefully analyzed, considering the potential signal artifacts generated by tDCS [80]. Functional near infrared spectroscopy (fNIRS) can be also used simultaneously with tDCS. An increase in the resting-state inter-hemispheric connectivity with increased flexion speed was measured after bi-hemispheric tDCS over the primary motor cortex [76]. tDCS over the sensorimotor cortex resulted in a significant reduction in the local brain activities required for the same sequential finger movement, representing a greater efficiency of neural transmission after tDCS [75]. With respect to simultaneous measurement with tDCS, fNIRS may be a better option than EEG, considering that its optical measurement system has no interference with the electrical current induced by tDCS. However, fNIRS has its limitations, such as difficulties associated with its applicability in hair-covered areas [81, 82] and its potential optical brain stimulation effect [83, 84].

**Fig. 4:**
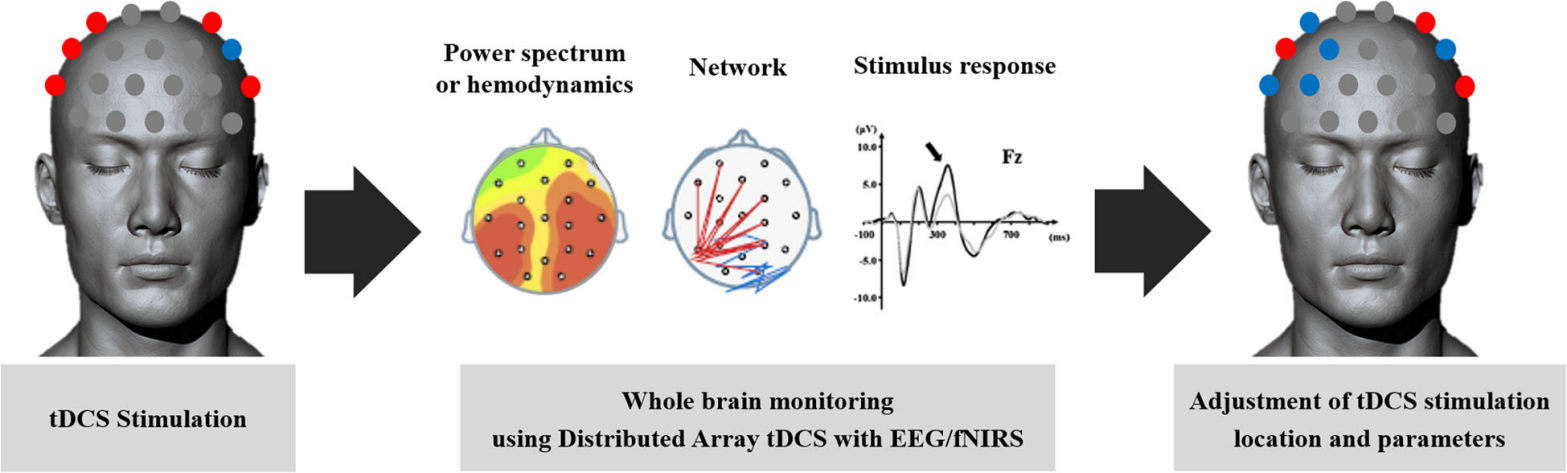
Potential response analysis after personalized tDCS combined with EEG or fNIRS. (A) EEG power spectrum, hemodynamics, functional network, and stimulus responses can be monitored within or near stimulation electrode areas in personalized electrode tDCS. (B) Those parameters can be monitored in the whole brain areas in distributed array tDCS combined with EEG of fNIRS. Red color represents anodes and blue color represents cathodes. tDCS: transcranial direct current stimulation; EEG: electroencephalography; fNIRS: functional near infrared spectroscopoy

Therefore, integrating EEG or fNIRS with tDCS may help the clinician to optimize the stimulation parameters that maximize the adaptive plasticity and recovery, despite their respective advantages and disadvantages. The schematic of a personalized tDCS, optimized by the potential real-time response analysis is shown in Fig. 4.

### Combination with task-oriented training using virtual reality

NIBS seems to be more effective when it is combined with task-oriented motor training. In previous animal stroke studies, combination of cortical stimulation and rehabilitation training induces brain plasticity and functional improvement [49, 85]. The beneficial effect of combination of NIBS with task-oriented training is also found in studies with stroke patients [86, 87]. Therefore, it may be important to combine task-oriented training with tDCS in clinical settings to optimize motor recovery after brain injury.

**Fig. 5:**
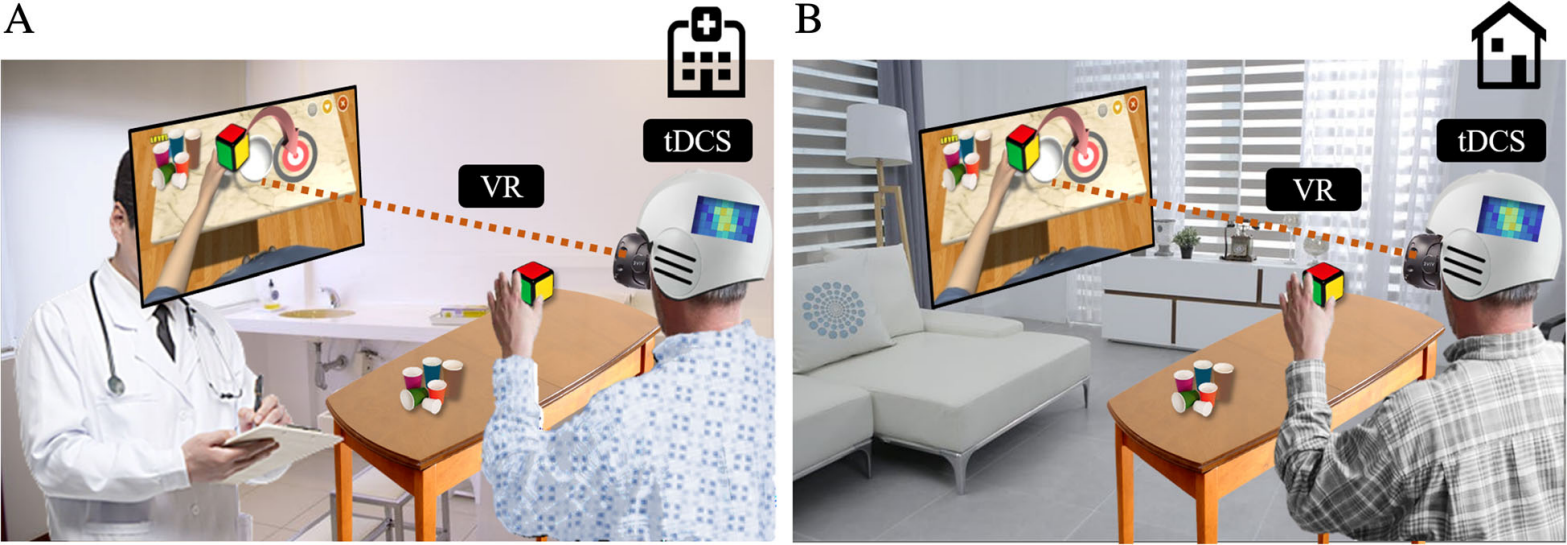
Merged system with tDCS and virtual reality. Patient with TBI can use this system in the hospital setting with the supervision of clinican (**a**) and can continue to use it at their home with tele-monitored system (**b**)

With respect to using modern technology, virtual reality (VR)-based rehabilitation can be a promising option. Task-oriented training can be provided using VR combined with tDCS. VR-based therapies can induce the repetitive task-oriented motions and may be beneficial to encourage patient motivation by gamifications and various interesting feedbacks [88–92]. In a recent Cochrane Systematic Review of the use of VR in stroke rehabilitation, it was found that when VR was used in combination with other usual care, there was improvement in the upper limb function (SMD 0.49, 95% CI 0.21 to 0.77, 210 participants from 10 studies), although the superiority to conventional therapy was not found [93]. In addition to the positive effects of VR alone, synergistic effects of combining VR with tDCS have been reported in stroke patients with motor impairment [94–97]. For example, Lee and Chen reported that a combination of tDCS and non-immersive virtual rehabilitation simultaneously was more effective than using each therapy alone in stroke patients with unilateral upper extremity weakness [94]. Therefore, merged system of tDCS and VR can provide a greater chance for recovery. In addition, tDCS and VR can be applied in the home setting due to its portability, relatively low cost, and possible tele-monitoring system, providing more time for rehabilitation [98, 99], which may contribute to better recovery (Fig. 5). Further studies are necessary to better investigate these possible benefits of combinational modalities.

## Conclusions

After TBI, tDCS can modulate the neuroplasticity and has the potential to promote motor recovery. Different changes in the brain at different times after the onset of TBI reveal the need for different neuromodulation approaches in accordance with the chronicity. Although many stroke studies have provided some implications of using tDCS in TBI for motor recovery, TBI is associated with different pathophysiology and more diffuse network disruptions; hence a well-designed clinical trial is needed in the future to prove the efficacy of tDCS and define the optimal stimulation parameters.

For more individualized approaches, imaging-based modelling or brain signal monitoring system can be combined with tDCS. By combining these technologies, optimal targeting may be possible, inducing a more favorable neuroplasticity. A combination of task-oriented training using a novel modern technology such as VR with tDCS can promote neuroplastic changes for motor recovery, which may lead to be a potent tele-rehabilitation tool in the home setting. Therefore, the development of a combination approach with tDCS and clinical trials to investigate the effect of this approach is required.

## Abbreviations

EEG: Electroencephalography
fMRI: Functional magnetic resonance imaging
fNIRS: Functional near infrared spectroscopy
MEP: Motor evoked potential
NIBS: Noninvasive brain stimulation
TBI: Traumatic brain injury
tDCS: Transcranial direct current stimulation
VR: Virtual reality
